# Advancing FAIR data towards comparable, organized, predictive AI-ready data for community validation

**DOI:** 10.1038/s42003-026-10694-y

**Published:** 2026-07-18

**Authors:** Elisha M. Wood-Charlson, Wolmar N. Åkerström, Lindsey Anderson, Mikayla A. Borton, Stephen K. Burley, Neil Byers, Ishwar Chandramouliswaran, Sylvain V. Costes, Paramvir S. Dehal, Mitchel Doktycz, Emiley Eloe-Fadrosh, Christopher Henry, Kjiersten Fagnan, Oliver Fiehn, Mahantesh Halappanavar, Bonnie Hurwitz, Marcin P. Joachimiak, Sean Jungbluth, Julia Koblitz, Gazi Mahmud, Lee Ann McCue, Thomas O. Metz, Nigel Mouncey, Christopher J. Mungall, Tiffanie M. Nelson, Valerie Skye, Amanda M. Saravia-Butler, V. Ratna Saripalli, Susannah G. Tringe, Tim Van Den Bossche, Adam P. Arkin

**Affiliations:** 1 Environmental Genomics and Systems Biology Division, E.O. Lawrence Berkeley National Laboratory, Berkeley, CA USA; 2 NBIS - National Bioinformatics Infrastructure Sweden, SciLifeLab, Uppsala University, Uppsala, Sweden; 3 Pacific Northwest National Laboratory, Richland, WA USA; 4 Department of Food Science and Human Nutrition, Colorado State University, Fort Collins, CO USA; 5 Rutgers, The State University of New Jersey, Piscataway, NJ USA; 6 DOE Joint Genome Institute, Lawrence Berkeley National Laboratory, Berkeley, CA USA; 7 Office of Data Science Strategy, National Institute of Health, Bethesda, MD USA; 8 Trivedi Institute for Space and Global Biomedicine, University of Pittsburgh, Pittsburgh, PA USA; 9 Biosciences Division, Oak Ridge National Laboratory, Oak Ridge, TN USA; 10 Computing, Environment, and Life Sciences Division, Argonne National Laboratory, Lemont, IL USA; 11 West Coast Metabolomics Center, University of California, Davis, Davis, CA USA; 12 BIO5 Institute, The University of Arizona, Tucson, AZ USA; 13 Estuary and Ocean Science Center, San Francisco State University, Tiburon, CA USA; 14 Leibniz Institute DSMZ-German Collection of Microorganisms and Cell Cultures, Braunschweig, Germany; 15 Australian BioCommons, The University of Melbourne, Melbourne, Victoria, Australia; 16 Amentum Services, Space Biosciences Division, NASA Ames Research Center, Moffett Field, CA USA; 17 Department of Biomolecular Medicine, Faculty of Medicine and Health Sciences, Ghent University, Ghent, Belgium; 18 VIB-UGent Center for Medical Biotechnology, VIB, Ghent, Belgium

**Keywords:** Microbiology, Computational biology and bioinformatics

## Abstract

The interrogation of data across biological and environmental systems has become increasingly complex. Fortunately, communities are adopting the FAIR (Findable, Accessible, Interoperable, Reusable) data principles for individual datasets, and continue to develop domain-specific, machine-actionable standards. However, integrating FAIR data for meta-analysis across data resources is still challenging. Understanding how disparate datasets are organized remains a manual, time-consuming process. Updating FAIR databases to reflect changes in knowledge is slow, allowing stale annotations and incorrect relationships to propagate, amplified by Artificial Intelligence (AI) systems that harvest data. Building on FAIR, we argue that data should be iteratively updated and improved. FAIR + COPE (Comparable, Organized, Predictive, Engaged) takes FAIR data and makes it Comparable, rapidly Organized (applying / updating standards) for Predictive models, which can be validated and improved by an Engaged community. We provide examples of FAIR + COPE resources and science use cases that highlight the importance of FAIR + COPE in scientific research.

## Abstract (web summary)

This Perspective explores how updating static, FAIR data/databases can lead to COPE: Comparable, Organized (AI-ready) data that supports novel Predictions of biological systems ready for human-in-the-loop validation by an Engaged community.

## Introduction

Scientific research requires a deep understanding of data to formulate and test predictions. As research questions become increasingly complex, integrating diverse data types and sources becomes necessary. For example, early microbial DNA sequencing efforts acknowledged the feat of publishing a single genome^1^. Now, undergraduate students regularly generate microbial genome publications^2–6^. Understanding biological systems, however, requires the integration of numerous, diverse data types that range in data frequency (discrete samples vs continuous sensor data), source (people, places, instruments, repositories, samples)^7–9^, and scale (space and time). Compiling complex datasets comes with challenges. The first - finding and accessing relevant data - has been made easier as data generators and repositories adopt the FAIR data principles (Findable, Accessible, Interoperable, and Reusable^10,11^). New curation tools and standards resources that support semantic integrations have emerged, as multidisciplinary research requires the combination of FAIR file-level standards with FAIR domain-specific, community-driven data standards, which also increases the AI-readiness of data^12^.

**Fig. 1:**
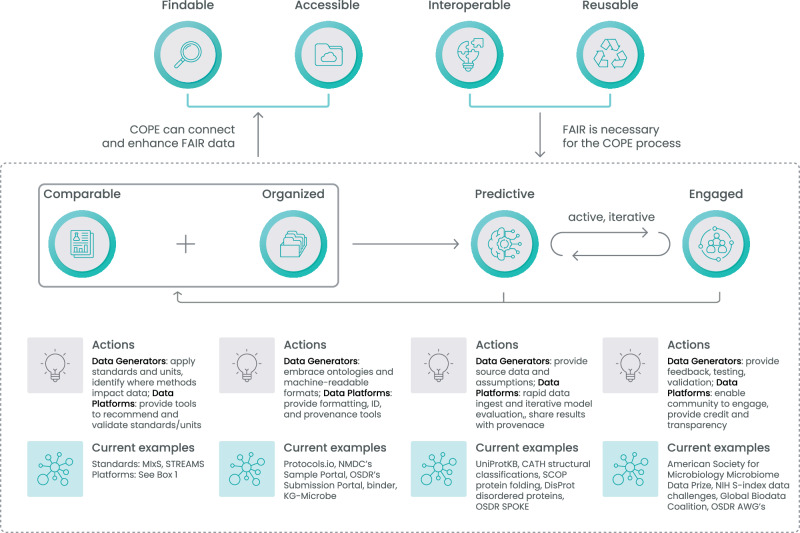
FAIR + COPE improvements to data enables iterative improvements to predictions that can be iteratively validated by the community. The FAIR data principles are necessary, but being able to make data Comparable and Organized for Predictive biology that can be validated by an Engaged community (FAIR + COPE) is an iterative process. Many resources support various aspects of FAIR + COPE, but the scientific community needs to invest in expanding and connecting those resources to fully enable the vision it embodies. Action and examples are provided to demonstrate feasibility.

However, challenges still exist. As described by Huttenhower et al^13^., the reuse and synergy in individual microbiome studies has many technicalities – assessing data quality, tracking data provenance, handling recalculation and versions of data products, and display and utilization of multiple, related analysis outputs. If we go beyond the FAIR principles, as they apply to static datasets, we still need to understand the complexity that underpins integration of many diverse, rapidly updating datasets. In brief, datasets must be made Comparable and Organized, per current standards and database versions, before they are ready for up-to-date, large-scale Predictive modeling, and then tested and validated by an Engaged community (Fig. 1). This must be an iterative process – as new knowledge is generated through testing and validation, updates to existing Comparable and Organized data need to be made and Predictive models need to be regenerated and re-evaluated. COPE (comparable, organized, predictive, and engaged) goes beyond being a set of principles; COPE acknowledges the active, iterative scientific process required to create and maintain the predictive inferences that act as the foundation of tools, parameters, and data products used for Artificial Intelligence (AI)-driven modeling and long-term data reuse.

### The COPE process for data integration goes beyond the FAIR principles

The FAIR principles are intentionally high-level, providing a foundation for data discoverability and access. In practice, however, FAIR compliance is not universal and does not guarantee that datasets from different sources can be directly integrated for domain-specific predictive modeling or AI-driven analyses. While FAIR enables the linking of data products by persistent identifiers, FAIR + COPE further extends that linking to additional domain-relevant metadata (e.g., sampling conditions, experimental methods, units, etc.^14^) for the evaluation of comparability and establishing organizational relationships. Currently, the COPE process is often manual, focused on a particular research question, and done without full transparency or reproducibility. Even if the data are FAIR, integration of domain-specific data across studies still requires harmonization. The data must be converted to comparable units and methodological parameters, and organized with consistent ontological labels and standardized terms. The resulting predictive analyses are often done on local machines, sometimes using older database or tool versions, and testing and validation is performed by close collaborators / co-authors already engaged in the project. This siloed approach also highlights how implicit predictive inference, such as gene annotation, normalization, and labeling, is often not well documented and difficult to update, therefore making data difficult to reuse.

COPE, as a mostly manual process, is time consuming, scope-limited, and hard to reproduce. To accelerate FAIR + COPE, application and assessment of units, parameters, standards, tools, and database updates must be automated, documented, and available for validation. Also, predictive assertions are not limited to analysis outputs of comparable, organized data; it also includes the necessary updates to labels or models that underpin what makes data comparable and organized. FAIR + COPE does not specify which predictive models should be used, only that the labels and units be made explicit, versioned, and retain a link to the original FAIR data they were derived from. Predictive requires Comparable data to define valid inputs, Organized structures to capture and propagate inferred relationships, and Engaged communities to iteratively evaluate, validate, and revise predictions as knowledge advances. This is what enables data from different origins to be confidently integrated, updated, and made ready for modeling and AI-workflows. Examples of automation using large language models (LLMs) already exist: leveraging FAIR-aligned workflows and tools to apply standardized labels to data (comparable) and rapidly identify contextually-related research outputs (organized), when labeled by standard identifiers^15,16^. By embedding domain-aware AI tools, FAIR can be transformed into FAIR + COPE, a more automated process that reduces effort while maximizing synergisms across diverse data sources and data origins to enable novel exploration and *Predictive* analysis. By leveraging comparable, organized data, metadata, and provenance, reproducible *Predictive* analyses can generate models of biological systems with accompanying estimates of uncertainty, or reveal integration issues and inconsistent analysis. These predictive model outputs are ready for human-in-the-loop testing and validation by the community, and the retained link to the source data, metadata, and provenance minimizes the data management effort required for data release or publication. Agent-assisted tools that support the initial steps in the FAIR + COPE processes will be essential for questions that extend beyond a single domain or institution; reach across time, space, and biological scales; and aim to integrate different data modalities. They will also accelerate the generation of AI-ready data.

### COPE is operationalized by integrating community engagement

FAIR has been embraced by the life sciences community^17^. However, once a FAIR dataset is released, it is rarely updated to incorporate new biological knowledge. FAIR + COPE embeds engagement into each step of Comparability, Organization, and Predictive analysis – from ontology curation and the formation of new standards^18^, to workflow sharing or open model validation – creating an iterative loop where data and models are continually improved (Fig. 1). Engagement in FAIR + COPE is operationalized through defined feedback structures between data producers, consumers, and the communities establishing standards. Examples of these feedback loops already exist: Data competitions, like the Critical Assessment of Metagenome Interpretation^19^, provide insight and user research opportunities, and community-led standards development and resource cataloging efforts^12,18,20–24^ that ensure data analysis and standards evolve alongside needs of the research. New resources continue to make discovery easier. For example, the Multi-Omics Metadata Standards Integration Working Group (MOMSI) surveyed the landscape of standards and generated a set of 250 standards, universal and omics specific, released as a collection (https://fairsharing.org/5742)^12^, with user exploration available at rda-momsi.github.io/Dashboard. Another example of deep community engagement was the development of the proposed nomenclatural code for uncultivated microbes, SeqCode^25,26^, and the widely utilized Genome Taxonomy Database (GTDB^27^; https://gtdb.ecogenomic.org), which provides consistently rank normalized genome-based taxonomy for prokaryotic genomes.

Engagement has always been a key component of the scientific process, typically at the beginning (proposal review) and the end of a study (publication review). FAIR + COPE elevates engagement as an essential component throughout the data lifecycle. The challenge will be to grow the existing culture of structured participation (i.e., review panels and journal reviews) into a more iterative, interactive culture, where creating and sharing comparable and organized data, evaluating diverse data sources for cross-study integration, and testing, validation, and updates of predictions from those data, is the norm. This is going to be even more critical in the age of AI, as human-in-the-loop validation and testing will be necessary to test AI-generated predictions. FAIR + COPE, as a process, enables the community to participate in the exploration of novel engagement strategies that are iterative, inclusive, measurable, and accountable.

### Ever increasing data complexity has already launched FAIR + COPE efforts

**Fig. 2:**
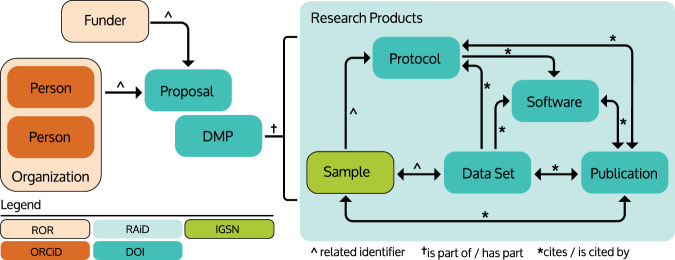
Persistent identifiers connect FAIR data to people, places, and other research outputs. Persistent Identifiers (PIDs) connect people, organizations, and research products, from individuals (ORCID) and organizations (ROR, Research Organization Registry) to samples (IGSN, International Geo/General Sample Number^34,53^). Related identifiers can be included in the PID metadata to establish connections in machine-readable formats. Data Management Plans (DMPs), especially those supported by the DMPTool (dmptool.org) help organize research outputs like protocols, datasets, software, and publications. These are typically given DOIs (Digital Object Identifier) and connected by relationship types, including is_part_of / has_part or cites / is_citedby. Finally, research projects can be given a Research Activity iDentifier (RAiD, https://raid.org).

Biological science has a long, successful history of data platforms and standards evolving with the needs of the community. In the 1980s, the International Nucleotide Sequence Database Collaboration (INSDC) was formalized to “capture and present the increasing volumes of sequence and annotation that arose from the emerging application of sequencing techniques”^28^ with a continual evolution of repository products from each member: National Center for Biotechnology Information (NCBI)^29^, European Molecular Biology Laboratory’s European Bioinformatics Institute (EMBL-EBI)^30^, and DNA Data Bank of Japan (DDBJ)^31^. Globally coordinated efforts for mass spectrometry-based proteomics (ProteomeXchange^32^) and metabolomics (Global Natural Products Social Molecular Networking^33^) have expanded support into multi-omics data. Since then, the growth in volume and complexity of data, the need for greater contextual framing (e.g., environmental, conditional), and the rapid technological advancements have been truly remarkable. The concept of a data platform has evolved from a static record archive towards more knowledge base platforms, where dynamic, machine-actionable FAIR data records contain details about its origin and links to sample(s)^34^ (important for the Nagoya Protocol and CARE principles^35^), sampling and laboratory protocols, related data products, the analysis software used for quality control or processing, and, finally, resulting scientific publications (Fig. 2). FAIR + COPE amplifies these integrated research products – expanding from static datasets to more interactive, cross-project modalities that support search, exploration, analysis, visualization, and publishing. Box 1 contains several representative FAIR + COPE data resources, many of which are affiliated with the authors of this manuscript. This list is not meant to be exhaustive, and we encourage the community to engage these projects as they continue to improve.

Box 1 Examples of FAIR + COPE resources recommended by the authors Australian BioCommons uses a nuanced engagement strategy (consultations, meetings, surveys) to understand and solve community-level data standards, analysis, and management challenges such as issues with data submission or access. Solutions – developed in partnership with infrastructures, institutes and repositories – include building or adopting technologies with ongoing community involvement and engagement. For example, Galaxy Australia’s Microbiology Lab (microbiology.usegalaxy.org.au) provides tools, workflows and compute for analysis, developed with the international microbial research community^54^ and the Australian Microbiome Analysis Community (www.biocommons.org.au/microbiomeanalysis).

Bacterial and Viral Bioinformatics Resource Center (BV-BRC^55^) leverages a unified data model to support a web-based AI assistant and enhanced web-based visualization and analysis tools as a service to aid in data integration for computable comparisons and workflows, which are shared back to the community for better understanding of pathogen biology and infectious diseases.

Department of Energy Systems Biology Knowledgebase (KBase^56^) has an object-based data model that enables researchers to (1) automatically convert data files into interoperable objects, (2) perform provenanced data analyses that build model predictions from complex, multiscale biological data, and then (3) immediately share with collaborators for feedback or publish them alongside a journal article.

ELIXIR^57^ maintains a list of endorsed deposition databases for the submission of experimental data and supports communities in converging on shared standards, databases, and tools within their domain (e.g. Microbiome Community and Federated Human Data Community)^58,59^. For example, the 1+ Million Genomes Framework (framework.onemilliongenomes.eu) offers a shared approach for secure, ethical, and interoperable genomic data sharing across Europe relying on common privacy and legal frameworks and use cases embodied in technical infrastructure and shared data models that will be implemented in a federated network of national nodes^60^.

Environmental System Science Data Infrastructure for a Virtual Ecosystem (ESS-DIVE) enables contributors to provide well-described and structured data through reporting formats and tools that were designed with community feedback and testing^61^. ESS-DIVE supports diverse Earth science (meta)data, including cross-domain metadata: dataset, location, and sample metadata; file-formatting guidelines: JSON, tab-deliminated, model data, etc; and domain-specific formats for biological, geochemical, and hydrological data types. Datasets containing file-level metadata and in csv formats are automatically parsed into a fusion database, which enables advanced search and discovery of data within files.

Global Biodiversity Information Facility (GBIF^62^) aggregates biodiversity data into a standardized taxonomic backbone, enabling species distribution and ecosystem models that are extended, validated, and refined through active community engagement.

International Nucleotide Sequence Database Collaboration (INSDC^28^) enforces structured sequence formats and standardized annotation pipelines, enabling integration of genomic data into countless research products, while continually expanding and updating the database through global community data submissions and curation.

MGnify^63^ deploys standardized, versioned analysis workflows that enable results to be interpreted across datasets, ensures provenance linking to sample metadata and raw data in INSDC, and incorporates community annotation updates, including enhanced sample metadata extracted by machine learning^64^.

UniProt Knowledgebase (UniProt^38^) provides access to important, curated data for understanding protein function and conservation. Curation of UniProtKB/Swiss-Prot is a highly engaged process, where experts use manual and ML techniques to review proposed annotations, and then update the UniProtKB database so that the knowledge is available to both humans and machines for further discovery.

National Microbiome Data Collaborative (NMDC^65^) provides sample handling and processing, standardized analysis workflows, and an interface for search and access, supported by a robust, flexible data schema that connects data across multi-omics data types and driven by community research questions.

Open Science Data Repository (OSDR^20^) contains experimental metadata linked to data files, processed data using publicly accessible pipelines to generate standardized, comparable data products, which are integrated into the visualization portal for reuse and comparison across studies.

Planet Microbe^66^ leverages ontology-driven links^66–68^ ‘omics data with the rich, contextual, environmental data collected. Spanning >2400 ocean samples over more than 10 years of global research cruises, Planet Microbe enables the exploration of geospatially- and temporally-resolved microbial community diversity and biogeochemical patterns. Predictive models explore the distribution, variability, and trajectory of global ocean processes and microbial community dynamics.

### Adoption of FAIR + COPE increases the value of scientific data

Adoption of FAIR + COPE can be incremental, and individual actions can improve the process of reliably integrating FAIR data over time. Here, we illustrate how we might transform a FAIR data meta-analysis into a FAIR + COPE process that supports microbial genotype-phenotype prediction, a process that can be updated to predict growth phenotypes of newly sequenced genomes or engineer strain optimization for target biomolecule chemical production. Full details regarding consideration involved in FAIR + COPE are discussed in the Supplemental Material.

#### Data integrated from multiple FAIR sources

For example: Microbial genomes with predicted genes and functional annotations (available from INSDC), growth × environment phenotype measurements (available for some genomes at BacDive^36^), gene essentiality data from RB-TnSeq experiments (available for a subset of genomes the Fitness Browser^37^), predicted homology groupings and genome similarity clusters (available at UniRef^38^), and taxonomic assignments via GTDB^27^.

#### Applying the COPE process to FAIR data

Comparable: Normalize growth measurements to common units (Units Ontology^39^ or Unified Code for Units of Measure) and conditions (Ontology of Microbial Phenotypes^40^, updated at Microbial Ecophysiological Trait and Phenotype Ontology - bioportal.bioontology.org/ontologies/METPO). Map gene functions to updated database release and shared identifiers. Standardize taxonomic labels to the current database release version. Note: mappings are also a prediction. Tool / version should be recorded.

##### Organized

Link genomes to genes to homology groups to functions. Link phenotype measurements to genomes to environmental conditions. Link gene essentiality calls to genes to phenotypes. Note: Record annotation pipelines with tools / versions.

##### Predictive

Gene calls, functional annotations, homology groupings, and taxonomy assignments are predictive assertions, linked to evidence and methods (tool / version). Metabolic models or ML tools apply phenotypes as predictions to genomes lacking measurements. Discrepancies between predicted and measured phenotypes are identified, highlighting where models have possible limitations.

##### Engaged

Community evaluation during, for example, a data competition identifies a misprediction and traces it to a misannotated gene family in the databases. A targeted experiment provides empirical evidence that supports revising the functional annotation, triggering an update to that annotation across the data portals.

However, these COPE process stages are not fully automated and remain fragmented (see examples below), and the platforms for engagement are limited in scope, awareness, and adoption. As a diverse set of co-authors, from many organizations and with a global representation, we aim to help realize a FAIR + COPE future. Success will be measured by the 1) increase of rapid, iterative application of the FAIR + COPE process, 2) more examples of community evaluation and exploration appearing as new standards or cross-domain ontological connections, 3) effective AI-assisted discovery of novel gaps in our current understanding of biology and environmental systems that can be experimentally validated, and 4) accelerated database updates that prompt existing predictive models to be rerun, or adjustment of model parameterization made, with explicit and transparent updates to confidence measurements.

As FAIR + COPE turns the FAIR principles into an iterative process (Fig. 1), we provide a generic process guide that outlines actions to operationalize COPE as a process, while also updating static FAIR datasets for AI integration, multi-study synthesis, and predictive modeling (Table 1). To check for consistency and completeness, future assessments of FAIR + COPE should model the existing FAIR assessment tools, i.e., FAIR Maturity Indicators^41^ or ELIXIR’s FAIR cookbook^42^, but extend to COPE-specific needs based on the recommendations in Table 1. Recommendation checks should document and validate: (i) the standard(s) or ontologies are registered with OBO Foundry^43^ and/or BioPortal^44^, (ii) the method of normalization or unit conversion, (iii) the method of uncertainty calculation, and (iv) provenance between physical samples, data, methods, and analytical workflows (discussed in detail in^34^), and their links to relevant predictive models (e.g., Fig. 2). Similar to FAIR data maturity self-assessments, review of FAIR + COPE assessments should verify that all steps were completed and reproducible. FAIR + COPE datasets and models should be rapidly discoverable and reused by research groups and organizations, with appropriate credit and attribution^45^. Below are two examples of how the FAIR + COPE process was applied during the research process, with excerpts from the authors on the manual process and overall impact.

**Table 1:** COPE extends the FAIR principles to capture the iterative, engaged process of science

	Recommendations	Resources	Engagement Strategies	FAIR + COPE Product
C	1. Express measurements using standardized units. Document conversions. 2. Document methodological processes (filters, kits, primers, thresholds) in sample metadata. 3. Follow FAIR principles: Apply ontology-based identifiers for common labels (environment, gene, chemical).	1. ISO, UO, UCUM 2. MIxS, STREAMS 3. ENVO, GO, ChEBI	Contribute missing terms to ontologies; share transformation scripts in public repositories; recommend standards to colleagues.	Comparable representations with explicit, re-executable mappings.
O	1. Capture provenance for data objects (collection methods, processing steps, software versions). 2. Express experimental design parameters in machine-readable formats. 3. Follow FAIR principles: Link related samples, protocols, derived datasets via persistent identifiers.	1-2. MIxS, STREAMS 3. RAiD, RO-Crate	Share data management plans in DMPtool; provide feedback / recommendations on data organization and application of persistent IDs.	Machine- readable provenance, experimental design, and cross-source relationships.
P	1. Link annotations / models / analyses to source data, state assumptions and parameters in machine-readable formats. 2. Report prediction confidence and uncertainty, provide links to evidence and validation datasets, highlight discrepancies. 3. Re-run models as source data / methods are updated. 4. Follow FAIR principles: Share outputs in reusable, machine-readable formats.	1-3. KBase, MGnify, ELIXIR 3. JSON-LD, RDF	Provide feedback on prediction errors; contribute validation datasets; flag outdated annotations.	Versioned predictive assertions linked to evidence, updates propagate as knowledge evolves.
E	1. Participate in community standards development, data curation, model validation activities. 2. Track feedback, acknowledge contributions, perform validation experiments, provide annotation updates with evidence. 3. Follow FAIR principles: Share metadata, scripts, and workflows; deposit in public repository with a DOI.	1. NMDC Ambassadors, OSDR AWGs, ELIXIR Communities, KBase UWGs 2-3. GitHub, KBase, Zenodo	Actively participate in ambassador programs and community working groups; comment on standards proposals; mentor or train peers.	Curated updates, corrections, and validation artifacts – attributed and versioned.

FAIR + COPE enables schema linking between data from disparate sources, updates and versioning of predictive assertions, and annotation that re-execute as knowledge evolves. COPE-compliant resources should steward these updates and coordinate community engagement activities to improve predictions and correct data errors.

#### Real-world demonstration of impact: Predicting bacterial phenotypic traits

Koblitz et al.^46^ provides a concrete example of how a published ML study can serve as a FAIR + COPE use case. The aim of the study was to predict bacterial phenotypic traits from genome-derived features at scale, using the BacDive knowledge base as the primary source of phenotype labels because it offers highly standardized strain-level data. Yet, despite this comparatively FAIR data starting point, making the dataset comparable and organized for modeling still required an extensive, largely manual COPE process. To organize the data and ensure diverse datasets were comparable, traits were preselected by data availability, genomes were filtered and linked to strains via consistent taxonomy identifiers and quality criteria, and ambiguous phenotype records (e.g., contradictory positive/negative labels across publications) were removed. The need for comparability became particularly visible for non-binary or mechanistically heterogeneous traits. For example, oxygen requirements – literature contradictions in BacDive meant that 7.1% of strains carried more than one oxygen state, making a multi-class approach unreliable and prompting re-organization into two separate models (AEROBE/ANAEROBE) with intermediate cases treated as negative and ambiguous cases excluded. For motility, an initial binary model failed systematically because most false predictions were gliding strains (89%), requiring iterative re-engineering of the input labels to align the target with the underlying biological mechanism. Even apparently straightforward continuous traits required explicit comparability decisions. Growth temperature as single measurements were mixed with temperature ranges, prompting additional filtering and careful definition of class boundaries.

Beyond what is described in the manuscript, and demonstrating the power of being engaged, was an additional exploratory attempt to predict pigmentation. Many strains were flagged as “pigment-producing” while the pigment name was recorded as “no pigment”, presenting an internal inconsistency that rendered the labels unsuitable for ML. The issue was reported and quickly corrected in the database. This illustrates exactly how FAIR + COPE is an iterative scientific investigation, and updating FAIR data saves time: controlled vocabularies and validation rules should enforce trait/value consistency (Comparable/Organized), and structured feedback loops between data users and curators (Engaged) turned FAIR data records into FAIR + COPE, AI-ready datasets that are reproducible and easier to validate.

#### Real-world demonstration of impact: Community river microbiome catalogue

Establishing FAIR + COPE processes at the onset of a multi-study cooperative project can streamline data integration and elevate the final product. The Genome Resolved Open Watershed database (GROWdb)^7^ started as a coordinated effort to align sampling designs, analytical workflows, and metadata reporting structures, while still allowing for individual researchers to pursue their research questions and hypotheses. By defining the minimum metadata, core environmental measurements, and quality control expectations, data comparability, and therefore aggregated analysis for statistical power, was possible beyond what is typically achieved by a single project effort. In addition, the GROWdb data were extensively labeled using standards and ontological terms that explicitly connect the microbes, as biological observations, to their environmental and ecosystem context. Sample-Data-Environment linkages enable rapid query, recombination, and direct use in modeling and ML workflows. Example predictions explored include: forecasting of microbial functional responses to changes in oxygen, nutrients, and hydrologic conditions; estimation of microbial contributions to biogeochemical fluxes across watersheds; and identification of conserved functional responses to shared environmental stressors. Throughout the collection, generation, and release of the GROWdb, community engagement was a key driver that refined both data quality and usability. For example, GROWdb leads aligned metadata practices with emerging community guidance for sample and metadata reporting^34^. This collaboration identified gaps, improved variable definitions, and resulted in more complete metadata, relative to what individual projects typically document. Engagement with scientists on specific projects such as lake-focused analyses that incorporated GROWdb into broader ecosystem studies (e.g., Fadum et al^47^.) further ensured which data structures would support diverse reuse cases. Although this engagement required additional coordination during data generation and curation, it ultimately reduced downstream effort by minimizing post hoc data cleaning and reinterpretation, resulting in a more consistent, extensible, and broadly reusable database.

GROWdb has already demonstrated impact through reuse by multiple research groups^48,49^, fostering new collaborations and enabling new studies beyond the original GROW dataset. These groups are using GROWdb to place site-specific observations and experiments into broader watershed and cross-system contexts. This illustrates how FAIR + COPE studies and databases can help microbiome research cross scales, from individual studies to regional synthesis.

These examples highlight how FAIR is necessary, but FAIR + COPE can operationalize making data directly usable for cross-study, model-driven science. FAIR + COPE allows researchers and organizations to build on their existing investments while increasing the scientific value of their data.

### Building towards a FAIR + COPE future

As a community of data generators, stewards, providers, and users across multiple institutions and infrastructures, we aim to manifest FAIR + COPE as a process. Some initial actions and solutions are outlined in Fig. 1. Critically, Engagement is not a principle – it is an embedded process within every stage of making data Comparable, Organized, and Predictive. Cross-dataset coordination, ontology alignment, and reproducible modeling all require sustained, structured collaboration between researchers, standards bodies, repositories, and tool developers. FAIR + COPE formalizes Engagement as a core operational process supported by measurable recommendations and strategies (Table 1). We acknowledge that implementing FAIR + COPE will not be easy. Data generation is already resource-intensive, and many existing COPE-ready resources (standards, ontologies, workflows) are not yet universally adopted. Yet, the effective application of standards have made transformational discoveries possible. E.g., Alphafold was possible because of standards established by the Protein Data Bank (PDB)^50^. By making Engagement explicit, we increase awareness and create training opportunities to support awareness and adoption of standards, such as NASA’s Open Science 101^51^, NASA’s Analysis Working Groups (AWGs), and the NMDC Ambassadors^52^. And, as a community, we can support FAIR + COPE by asking funders, organizations, and publishers to: (1) support the coordination of FAIR data across platforms; (2) fund co-development of tools that make compliance easier; (3) provide free, open-source training and resources; and (4) formally recognize and reward researchers for both producing and improving FAIR + COPE datasets. Adding FAIR + COPE to our research culture will enable humans and machines to more reliably assemble and explore integrated data toward actionable insights.

## Supplementary information

Supplementary Information

Transparent Peer Review file
