# Supplementary material for: Advancing FAIR data towards comparable, organized, predictive AI-ready data for community validation
Source: Commun Biol. 2026 Jul 18;9:983. doi: 10.1038/s42003-026-10694-y (PMC13380610; doi:10.1038/s42003-026-10694-y)
Supplement: Supplementary file 1 — Supplementary Information [file 42003_2026_10694_MOESM1_ESM.pdf]

Supplementary Discussion: Advancing FAIR data towards comparable, organized, predictive AI-ready data for community validation

FAIR+COPE for integration of complex biological data and analyses

Extraction and translation of information from data and associated metadata is often a manual and subjective task, especially if the files contain non-standardized values/terms. This is complicated by the number of discrete, highly specialized data portals, which are often not linked, don't always leverage the same labels (e.g., NCBI taxonomy vs GTDB taxonomy, may not have sufficient resources for curation, may have missing or boutique labels, and/or may not have long-term support and sunset after a few years.

Comparable data define the scope of analysis

Determining the comparability of datasets from multiple sources is time-consuming but necessary for performing integrative analyses. Scientists must assess whether datasets are truly comparable, and if necessary, transform them (e.g., normalization, unit harmonization). This problem is subtle and often question-specific, but can be broken down into several concepts that can mostly be addressed by applying domain-specific data stewardship.

Ontological Comparability: Data must be of the same ontological type – as observations about the same entity or class of entities/ For example, chemical concentrations, species abundances, or water flow rates can be compared within their types, even at different levels, as long as those levels have defined relationships. For example, different species abundance measurements can be adjusted and aggregated at the same taxon level (i.e., all species are within a single genus, but not all genus members are of the same species) because taxonomies are nested hierarchies. However, comparable abundance measures have other considerations.

Contextual Compatibility: Data must have a compatible context – as ontological comparability may still be limited by context of data collection. For instance, chemical concentrations in milligrams (mg) per liter (L) of water are not comparable to those expressed in mg per kilogram (kg) of sediment, as they refer to different environments and have incompatible units. However, latitude/longitude can be converted by a mathematical relationship, and used to place samples within an ecosystem (using the ENVO) or enhance the metadata to capture elevation (in meters).

Methodological Bias: Data are biased by collection/processing techniques. For example, species abundance datasets are not comparable if they use different thresholds to observe organisms. Abundance counts of organisms in 10μm and 0.2μm filtered water do not capture the same community of organisms. Other examples could be expeditions of different duration (1 day versus 1 month), type of kit used for nucleic acid extraction (batch effect), use of different primer sequences for target gene amplification, or even shallow vs deep sequencing of samples. Finally, combining datasets with differing precision and accuracy is limited by the least precise and accurate measurements (as in Ontological Comparability).

Scaling efforts to integrate data across multiple resources necessitates methods for assessing and articulating the degrees of above comparisons in composite datasets, as it is important to understand the limitations set by the combined data. Immediate access to the details required to assess data comparability enables more effective integration and analysis of diverse datasets.

Organized data enable rapid exploration and analysis

Once data are determined comparable, data organization is next. Systematic organization can enable rapid exploration and assessment and establish baseline models for statistical analysis (e.g., experimental design) and modeling (e.g., causal relationships).

Categorization: Data with ontological labels and units aids in grouping data that observe the same entity (e.g., 10μm filtered seawater or sediment chemistry in mg/L) for direct comparison.

Concept: Data exists within an experimental framework. Capturing parameter design, controls, replicates, and the purpose behind each measurement helps explain the context and intent behind the data. This organization is typically reported in the text of the publication. Recent improvements in text mining has enabled rapid conversion of experimental design and statistical analyses into machine-readable formats, and lead to the generation of novel databases for targeted questions (e.g., 15,000 metabolic engineering targets across microbial hosts, doi: 10.64898/2025.12.15.694291) enhancing data reuse capabilities.

Connection: No data exists in isolation. Declaring those physical and causal relationships informs how we model dynamic systems, analyze interactions, and explore complex networks. Spatial relationships (e.g., anatomical or geographical proximity) and temporal sequencing in sample design, chemical reactivity of environment, and evolutionary lineage of community members are connections that can be inferred with proper categorization and context. Acknowledging that relationships are complex, additional considerations or novel experimental frameworks may arise when disparate data are brought together (even with good metadata).

Provenance: Understand the origin of data. Document collection/processing protocols, devices, reagents (including lot numbers), and instruments used for collection, and tools (including versions) used to process data, to assess when, how, and what comparisons are possible. Provenance also enables tracking of data reuse, which enhances transparency, supports reproducibility, ensures credit and recognition, and promotes open science.

Categorization, conceptual framing, and *provenance* are part of the deeper exploration into the "IR" in FAIR. *Connection* places the data back in a working model of the universe from which the physical samples and data originated. A single model is unlikely to reasonably capture all applications or data spaces. However, ensuring data are comparable and organized across repositories and knowledge bases allows for those data to be accurately integrated into pre-existing data models; thereby enhancing the predictability of the models and enabling cross-discipline collaborations to begin exploring and advancing our understanding of complex systems.

Predictive capabilities refine and improve data coherence

Prediction and inference ultimately test our understanding of a system, and are essential for scaling knowledge to countless variants that are infeasible or impossible to measure directly. Prediction extends comparability and organization by generating new, knowledge-driven relationships. Predictions may shift with new data or improved algorithms. Initial predictions may have lower confidence, but will improve with additional data and re-evaluation, and integration of diverse data sources may strengthen predictions. Predictions generate hypotheses and identify new comparisons possible across integrated data resources.

As part of the FAIR+COPE process, prediction both enhances and evaluates comparability and organization, revealing integration issues or incomplete/inconsistent analyses. Predictions and inferences should employ standardized practices for documentation, evidence association, and confidence assessment. Different methods may produce conflicting predictions; therefore, robust predictions require an understanding of the quality, uncertainty, and source (causation or correlation) of the underlying data. Methods to propagate uncertainty and detect inconsistencies must be established and applied. Analysts should have access to accurate metadata (provenance) to track assertions, and be able to formally and transparently validate predictions and suggest improvements.

Engaged community is needed to explore, experiment, and share

The COPE+FAIR process would not be an iterative feedback loop without an engaged community providing feedback and validation, expanding standards and ontologies, and contributing design ideas for data platform improvement. We also regularly use data and conclusions generated by others, and ask research questions that cross several research domains (biogeochemical cycles, weather and ecosystem models, landscape changes at scale, etc.). Most of the data we use are derived data products that come with inherent bias: when, where, why, and how data were collected; assumptions built into analysis tools; and the lens through which we view the results influence how we interpret the data. As we collectively evaluate and refine predictive models into the future, we will increasingly ask for assistance from AI agents. It will be crucial for FAIR+COPE, as a process, to be open and transparent, with humans in the loop at every stage. AI-assisted FAIR+COPE will improve community feedback mechanisms (while retaining credit for contributions), and accelerate the iterative nature of the process by rapidly identifying areas of low confidence or gaps in knowledge, for additional research and data that will improve the models over time.
