# Supplementary material for: Advancing FAIR data towards comparable, organized, predictive AI-ready data for community validation
Source: Commun Biol. 2026 Jul 18;9:983. doi: 10.1038/s42003-026-10694-y (PMC13380610; doi:10.1038/s42003-026-10694-y)
Supplement: Supplementary file 2 — Transparent Peer Review file [file 42003_2026_10694_MOESM2_ESM.pdf]

nature portfolio

Peer Review File

Advancing FAIR data towards comparable, organized, predictive AI-ready data for community validation

Corresponding Author: Professor Adam Arkin

This file contains all reviewer reports in order by version, followed by all author rebuttals in order by version.

Version 0:

Reviewer comments:

Reviewer #1

(Remarks to the Author)
This paper focuses on the challenges of data reuse, and the proposed COPE process seems to provide a useful framework for thinking about how to do this and for implementing it in the authors' research domain.

The authors claim that the FAIR principles apply to individual datasets only, and that it is important to compare datasets across studies and data resources. Therefore we need something more than the FAIR principles. However, this is incorrect. The whole purpose of FAIR is to ENABLE such reuse and integration. Datasets can and should be combined, and assessing whether that is possible is easier if those datasets are FAIR. Datasets that have been combined can themselves also be represented following the FAIR principles. There are infrastructure frameworks that specifically support these processes already in existence (e.g. see this work on FAIR Digital Objects and Research Object Crates (ROCrates) https://pubmed.ncbi.nlm.nih.gov/38217405/ - I am not involved in this work, I just think it is a good example of what can be done with integrated datasets and FAIR). How does COPE compare to these other processes and frameworks?

The authors also claim that FAIR is generic and not domain specific. However, the Reusability principles clearly state that FAIR data should use domain-specific standards. If these standards are insufficient, they should be further developed and improved, but that is maybe why the implementation of FAIR has been more successful in some domains than others.

The reusability principles of FAIR are as follows:

R1. (Meta)data are richly described with a plurality of accurate and relevant attributes

R1.1. (Meta)data are released with a clear and accessible data usage license

R1.2. (Meta)data are associated with detailed provenance

R1.3. (Meta)data meet domain-relevant community standards

The idea of COPE is good, but the framing of COPE by the authors seems to involve misrepresenting the FAIR principles. This is not necessary and weakens the arguments given by the authors. COPE is an interesting way of operationalsing data reuse. If the data is FAIR, it is easier to perform the COPE process. That is the relationship as I see it between FAIR and COPE.

The authors include some real world examples of FAIR-COPE, but no assessment of how FAIR these collections are. I see they link to zenodo records, but not to curated public repositories. How easy is it to dynamically update these records with new knowledge (one of the stated key reasons for developing COPE)

The authors list a number of COPE supporting resources. Is this the assessment of the authors? Or did these resources endorse COPE and declare themselves COPE supporting resources? It would be more transparent to explicitly state this in the manuscript.

Reviewer #2

(Remarks to the Author)
This is an important topic for entire scientific community, although genomics has been spurring efforts in this area for 20 or more years now. Many researchers, programs and institutions are getting behind the FAIR principles, but not all of them understand the importance of developing resources to implement interoperability and data reusability in a valuable way. This paper has several sections that help convey that message to the community. It probes what it means to have a deep level data interoperability (conducive to integration). It (correctly, in my opinion) points out the role data reuse for prediction can and should play to help advance models and standards for data. It also advocates a community-based approach to promoting resources to make data more interoperable.

Whole manuscript: Are the supplementary materials referenced in the main text somewhere? I didn't see they were...

line 140: "And new resources are available to make discovery easier." Sentence fragment
line 175: "and finally resulting ..." add commas (and, finally, resulting...)
line 178: "Box 1 contains several of FAIR+COPE data resources..." Consider "contains several representative FAIR+COPE data resources..."
line 236: Planet Microbe This is a huge, run-on sentence... Break up.

Version 1:

Reviewer comments:

Reviewer #1

(Remarks to the Author)

The revised version of the manuscript represents FAIR well and makes very good arguments about why this community also needs COPE to enhance the value and worth of FAIR over the longer term.

There is nothing static about the FAIR principles. It is perfectly possible to have multiple versions of the same FAIR dataset. However, for many data repositories that accept data submitted by the community, FAIR is implemented in a static way and it can impede reuse. COPE can play an important role in addressing this problem, as well as highlighting the importance of domain-specific standards and ontologies.

I think this version of the paper is much improved and contains interesting contributions for the research community.

Open Access This Peer Review File is licensed under a Creative Commons Attribution 4.0 International License, which permits use, sharing, adaptation, distribution and reproduction in any medium or format, as long as you give appropriate credit to the original author(s) and the source, provide a link to the Creative Commons license, and indicate if changes were

made.
In cases where reviewers are anonymous, credit should be given to 'Anonymous Referee' and the source.
The images or other third party material in this Peer Review File are included in the article's Creative Commons license, unless indicated otherwise in a credit line to the material. If material is not included in the article's Creative Commons license and your intended use is not permitted by statutory regulation or exceeds the permitted use, you will need to obtain permission directly from the copyright holder.
To view a copy of this license, visit https://creativecommons.org/licenses/by/4.0/

Reviewers' and Editor comments:

Given that the original Reviewer #3 from Nature Microbiology was unavailable, the editors personally assessed this part of the rebuttal. We believe that all of Reviewer #3's points were addressed, with the exception of:

“Finally, as with many data centric approaches, one thing is missing, and that is the links to the ‘physical’ samples. All too often the link between data and physical sample is broken. While many folks consider that lodging a sequence onto NCBI or ENA will suffice, the ‘ultimate’ approach would undoubtedly involve keeping a link between data and the sample itself. While is not practical or economic to keep every sample, for those samples important to scientific research, it would provide the ultimate gold standard, allow reproducibility and improve the confidence and stringency of the work we do. It would be nice to see at the very least, an acknowledgement of the potential importance of the role of biobanks and culture collections, particularly the link between provenance, sample, metadata and links to genome information. I did find a link online to agmicrobiomebase/data which attempts to do this (it links out to ENA and include a notable soil dataset), although it is far from perfect it does seek to address some of the issues.”
(As it is unclear how/where biobanks or physical samples were elaborated on in the current manuscript.)

Noted where physical samples connections are discussed in a recent manuscript (shares several co-authors)

Finally, please avoid:
-Any punny uses of “COPE” throughout the text.
Ex. Line 75: “we still need to COPE with the complexity” > “we would need COPE to understand the complexity” (or similar, though this sentence also appears before the acronym is formally introduced)

Thanks for the suggestion - fixed as recommended.

-Using italics for emphasis:
Ex. Lines 338-339: “We reported the issue, and it was quickly corrected in the database”

All italics have been removed from the text.

Reviewer #1 (Remarks to the Author):

This paper focuses on the challenges of data reuse, and the proposed COPE process seems to provide a useful framework for thinking about how to do this and for implementing it in the authors' research domain.

The authors claim that the FAIR principles apply to individual datasets only, and that it is important to compare datasets across studies and data resources. Therefore we need something more than the FAIR principles. However, this is incorrect. The whole purpose of FAIR is to ENABLE such reuse and integration. Datasets can and should be combined, and assessing whether that is possible is easier if those datasets are FAIR. Datasets that have been combined can themselves also be represented following the FAIR principles. There are infrastructure frameworks that specifically support these processes already in existence (e.g. see this work on FAIR Digital Objects and Research Object Crates (ROCrates) https://pubmed.ncbi.nlm.nih.gov/38217405/ - I am not involved in this work, I just think it is a good example of what can be done with integrated datasets and FAIR). How does COPE compare to these other processes and frameworks?

Thanks for these comments - we absolutely agree. Now 10 years beyond the FAIR data principles publication, the infrastructure / standards community is starting to enable more effective strategies to extend FAIR, including ROcrate and RAiDs. However, these are both still nascent efforts that have not been fully adopted by the community (yet!?). The goal of FAIR+COPE is to emphasize that, while things are getting easier, no one is really discussing how to keep on top of rapid underlying changes to knowledge, and how that impacts the interpretation of FAIR datasets, which are currently not, to our knowledge, re-annotated or updated in a consistent manner. Take microbial taxon labels – the community has 2 options (NCBI and GTDB), which are updated at different times / rates, and the mapping file between them is not 1-1 because of challenges in taxon resolution, etc. The authors wanted to declare the need for broader awareness and discussion of how to rapidly do consistent, federated updates to FAIR data, especially in the age of AI.

The authors also claim that FAIR is generic and not domain specific. However, the Reusability principles clearly state that FAIR data should use domain-specific standards. If these standards are insufficient, they should be further developed and improved, but that is maybe why the implementation of FAIR has been more successful in some domains than others.

We absolutely agree with this! While also noting that the FAIR publication does state, “FAIR differs in that it describes concise, domain-independent, high-level principles that can be applied to a wide range of scholarly outputs.” In addition, the co-authors’ have noticed that, even with higher adoption rates in the life sciences community, we regularly encounter members of our community that have not yet heard of the FAIR data principles (10 years on), let alone domain specific standards. While the domain-specific standards are always improving, people can’t use them if they aren’t aware of them, let alone know how to apply them effectively.

The reusability principles of FAIR are as follows:

R1. (Meta)data are richly described with a plurality of accurate and relevant attributes

R1.1. (Meta)data are released with a clear and accessible data usage license

R1.2. (Meta)data are associated with detailed provenance

R1.3. (Meta)data meet domain-relevant community standards

The idea of COPE is good, but the framing of COPE by the authors seems to involve misrepresenting the FAIR principles. This is not necessary and weakens the arguments given by the authors. COPE is an interesting way of operationalsing data reuse. If the data is FAIR, it is easier to perform the COPE process. That is the relationship as I see it between FAIR and COPE.

We appreciate the reviewer's comment here - we are trying to get to the same place! However, we interpret the principles a bit more cautiously. For example, from the FAIR publication:

> "The optimal state—where machines fully 'understand' and can autonomously and correctly operate-on a digital object—may rarely be achieved. Nevertheless, the FAIR principles provide 'steps along a path' toward machine-actionability; adopting, in whole or in part, the FAIR principles, leads the resource along the continuum towards this optimal state. In addition, the idea of being machine-actionable applies in two contexts—first, when referring to the contextual metadata surrounding a digital object ('what is it?'), and second, when referring to the content of the digital object itself ('how do I process it/integrate it?'). Either, or both of these may be machine-actionable, and each forms its own continuum of actionability."

And…

> "Creating bespoke parsers, in all computer languages, for all data-types and all analytical tools that require those data-types, is not a sustainable activity. As such, the focus on assisting machines in their discovery and exploration of data through application of more generalized interoperability technologies and standards at the data/repository level, becomes a first-priority for good data stewardship."

We commend the FAIR authors for their foresight! Advances in AI are finally making the transition along the continuum of actionability more plausible. We describe in the manuscript how AI tools are being used to help apply metadata standards to datasets. However, datasets and databases still have to be kept up-to-date for FAIR+COPE to work.

We have updated some language in the text to more explicitly lay out this context (as the technology has progressed significantly since the last review).

The authors include some real world examples of FAIR-COPE, but no assessment of how FAIR these collections are. I see they link to zenodo records, but not to curated public repositories. How easy is it to dynamically update these records with new knowledge (one of the stated key reasons for developing COPE)

Authors for both examples, BacDive phenotype traits and GROW river genome database, were asked to join this publication explicitly because they did the extra effort to FAIR+COPE their datasets. BacDive is a curated public repository, which was updated / corrected because of this

published example. GROW has their data available in numerous curated resources - NCBI, NMDC, KBase, etc.

The authors list a number of COPE supporting resources. Is this the assessment of the authors? Or did these resources endorse COPE and declare themselves COPE supporting resources? It would be more transparent to explicitly state this in the manuscript.

Added language to emphasize that the FAIR+COPE resources are offered to the community by the co-authors.

Reviewer #2 (Remarks to the Author):

This is an important topic for entire scientific community, although genomics has been spurring efforts in this area for 20 or more years now. Many researchers, programs and institutions are getting behind the FAIR principles, but not all of them understand the importance of developing resources to implement interoperability and data reusability in a valuable way. This paper has several sections that help convey that message to the community. It probes what it means to have a deep level data interoperability (conducive to integration). It (correctly, in my opinion) points out the role data reuse for prediction can and should play to help advance models and standards for data. It also advocates a community-based approach to promoting resources to make data more interoperable.

Whole manuscript: Are the supplementary materials referenced in the main text somewhere? I didn't see they were…

Apologies! That reference was missing from the text.

line 140: "And new resources are available to make discovery easier." Sentence fragment

Fixed

line 175: "and finally resulting ..." add commas (and, finally, resulting...)

Fixed

line 178: "Box 1 contains several of FAIR+COPE data resources..." Consider "contains several representative FAIR+COPE data resources..."

Fixed

line 236: Planet Microbe This is a huge, run-on sentence... Break up.

Fixed
